# Differential Response of Immunohistochemically Defined Breast Cancer Subtypes to Anthracycline-Based Adjuvant Chemotherapy with or without Paclitaxel

**DOI:** 10.1371/journal.pone.0037946

**Published:** 2012-06-05

**Authors:** George Fountzilas, Urania Dafni, Mattheos Bobos, Anna Batistatou, Vassiliki Kotoula, Helen Trihia, Vassiliki Malamou-Mitsi, Spyros Miliaras, Sofia Chrisafi, Savvas Papadopoulos, Maria Sotiropoulou, Theodoros Filippidis, Helen Gogas, Triantafyllia Koletsa, Dimitrios Bafaloukos, Despina Televantou, Konstantine T. Kalogeras, Dimitrios Pectasides, Dimosthenis V. Skarlos, Angelos Koutras, Meletios A. Dimopoulos

**Affiliations:** 1 Department of Medical Oncology, “Papageorgiou” Hospital, Aristotle University of Thessaloniki School of Medicine, Thessaloniki, Greece; 2 Laboratory of Biostatistics, University of Athens School of Nursing, Athens, Greece; 3 Laboratory of Molecular Oncology, Hellenic Foundation for Cancer Research, Aristotle University of Thessaloniki School of Medicine, Thessaloniki, Greece; 4 Department of Pathology, Ioannina University Hospital, Ioannina, Greece; 5 Department of Pathology, Aristotle University of Thessaloniki School of Medicine, Thessaloniki, Greece; 6 Department of Pathology, Metaxas Cancer Hospital, Piraeus, Greece; 7 First Department of Surgery, “Papageorgiou” Hospital, Aristotle University of Thessaloniki School of Medicine, Thessaloniki, Greece; 8 Department of Pathology, “Hygeia” Hospital, Athens, Greece; 9 Department of Pathology, “Alexandra” Hospital, Athens, Greece; 10 “Micromedica” Histopathology Laboratory, Athens, Greece; 11 First Department of Medicine, “Laiko” General Hospital, University of Athens School of Medicine, Athens, Greece; 12 First Department of Medical Oncology, “Metropolitan” Hospital, Piraeus, Greece; 13 Translational Research Section, Hellenic Cooperative Oncology Group, Data Office, Athens, Greece; 14 Second Department of Internal Medicine, Oncology Section, “Hippokration” Hospital, Athens, Greece; 15 Second Department of Medical Oncology, “Metropolitan” Hospital, Piraeus, Greece; 16 Division of Oncology, Department of Medicine, University Hospital, University of Patras School of Medicine, Patras, Greece; 17 Department of Clinical Therapeutics, “Alexandra” Hospital, University of Athens School of Medicine, Athens, Greece; Health Canada, Canada

## Abstract

**Background:**

The aim of the present study was to investigate the efficacy of adjuvant dose-dense sequential chemotherapy with epirubicin, paclitaxel, and CMF in subgroups of patients with high-risk operable breast cancer, according to tumor subtypes defined by immunohistochemistry (IHC).

**Materials and Methods:**

Formalin-fixed paraffin-embedded (FFPE) tumor tissue samples from 1,039 patients participating in two adjuvant dose-dense sequential chemotherapy phase III trials were centrally assessed in tissue micro-arrays by IHC for 6 biological markers, that is, estrogen receptor (ER), progesterone receptor (PgR), HER2, Ki67, cytokeratin 5 (CK5), and EGFR. The majority of the cases were further evaluated for HER2 amplification by FISH. Patients were classified as: luminal A (ER/PgR-positive, HER2-negative, Ki67^low^); luminal B (ER/PgR-positive, HER2-negative, Ki67^high^); luminal-HER2 (ER/PgR-positive, HER2-positive); HER2-enriched (ER-negative, PgR-negative, HER2-positive); triple-negative (TNBC) (ER-negative, PgR-negative, HER2-negative); and basal core phenotype (BCP) (TNBC, CK5-positive and/or EGFR-positive).

**Results:**

After a median follow-up time of 105.4 months the 5-year disease-free survival (DFS) and overall survival (OS) rates were 73.1% and 86.1%, respectively. Among patients with HER2-enriched tumors there was a significant benefit in both DFS and OS (log-rank test; p = 0.021 and p = 0.006, respectively) for those treated with paclitaxel. The subtype classification was found to be of both predictive and prognostic value. Setting luminal A as the referent category, the adjusted for prognostic factors HR for relapse for patients with TNBC was 1.91 (95% CI: 1.31–2.80, Wald's p = 0.001) and for death 2.53 (95% CI: 1.62–3.60, p<0.001). Site of and time to first relapse differed according to subtype. Locoregional relapses and brain metastases were more frequent in patients with TNBC, while liver metastases were more often seen in patients with HER2-enriched tumors.

**Conclusions:**

Triple-negative phenotype is of adverse prognostic value for DFS and OS in patients treated with adjuvant dose-dense sequential chemotherapy. In the pre-trastuzumab era, the HER2-enriched subtype predicts favorable outcome following paclitaxel-containing treatment.

## Introduction

Breast cancer is the most common female malignancy in the USA accounting for 27% of all new cancer cases, although its yearly incidence is declining since 1999 [1]. Anthracyclines (doxorubicin and epirubicin) and taxanes (paclitaxel and docetaxel) are the most commonly used chemotherapeutic agents in the adjuvant treatment of “high-risk” breast cancer. The Early Breast Cancer Trialists' Collaborative Group (EBCTCG) recently presented compelling data showing that taxane-based regimens are superior to anthracycline-based regimens [2], even though their superiority has been confronted by the results of the recently published “Taxotere as Adjuvant Chemotherapy Trial” (TACT) [3]. Consequently, the role of taxanes in the adjuvant setting remains equivocal (reviewed in ref. [4]). It should be noted however, that two recent meta-analyses of adjuvant chemotherapy trials, with or without taxanes, have shown a clear taxane benefit in high-risk early breast cancer patients [5], [6]. The combination of an anthracycline with a taxane seems therefore to be a logical step forward in the treatment of early “high-risk” breast cancer, since their mechanism of action is different and are not cross-resistant [7]–[10]. However, their optimal sequence of administration, i.e. concurrently or sequentially, and the role of dose intensity is not well defined yet. The sequential approach may be more effective, since it allows drugs to be given at full doses with less frequent dose reductions, thus increasing dose intensity (DI). Nevertheless, in the recently published Breast Cancer International Research Group-005 (BCIRG-005) study the sequential administration of doxorubicin and cyclophosphamide followed by docetaxel did not improve the outcome of patients compared to the concurrent administration of the three drugs [11].

Dose-dense chemotherapy (the increase of DI by reducing the interval between cycles), with the use of granulocyte colony stimulating factors (G-CSFs), is the result of the application of mathematical models of cell growth kinetics into the clinical practice [12]. This type of chemotherapy has been successfully evaluated during the last 15 years in patients with operable breast cancer (reviewed in [13]–[15]), even in those of older age [16].

Dose-dense sequential chemotherapeutic regimens are considered highly effective and better tolerated than conventional regimens in the adjuvant breast cancer setting [17]. In line with the enthusiastic embracement of the “dose-dense and/or sequential” concept in the design of randomized studies adopted by the majority of collaborative groups, including ours, two randomized phase III trials in patients with high-risk operable breast cancer were designed and conducted sequentially by the Hellenic Cooperative Oncology Group (HeCOG). In the first of these trials (HE10/97) we compared dose-dense sequential chemotherapy with epirubicin and CMF (cyclophosphamide, methotrexate and 5-fluorouracil), with or without paclitaxel [18]. We did not demonstrate a benefit in disease-free survival (DFS) or overall survival (OS) between the two groups of patients, however the study was not powered to identify a subtle but still biologically meaningful difference. Conversely, the recently published Docetaxel Epirubicin Adjuvant (DEVA) trial [19] has shown that the substitution of docetaxel by epirubicin for the last three cycles of treatment improved both DFS and OS in postmenopausal patients with node-positive early breast cancer, compared to six cycles of epirubicin monotherapy. In the second trial (HE10/00), concurrent administration of epirubicin and paclitaxel was compared with dose-dense sequential administration of the drugs. Both regimens were followed by dose-dense CMF [20], [21]. No difference was observed in DFS and OS between the two treatment groups [21], however the concurrent arm had increased toxicity [20]. Similarly, in the BCIRG-005 trial mentioned above [11], the two regimens were equally effective but differed in their toxicity profile, as well.

Of note, these randomized trials share with other similar trials a major methodological and biological disadvantage in their design. They simply include all patients fulfilling common clinico-pathological criteria and breast cancer is treated as a single clinical entity. However, during the past decade, DNA-microarray studies have identified five molecularly discrete subtypes of breast cancer, with different response to chemotherapeutic agents and distinct prognosis [22], [23]. These seminal studies, even though small in size, clearly showed the path that clinicians had to follow to successfully deal with breast cancer heterogeneity, using molecular tools for treatment decisions. Nevertheless, these high-throughput technology assays are not easy to be applied in large trials mainly because they are costly and require fresh tumor tissue that is not available in most studies.

To overcome these hurdles, investigators have recently been using formalin-fixed paraffin-embedded (FFPE) tumor tissue to immunohistochemically define breast cancer subtypes, which markedly resemble those defined by gene expression analysis and are at least as accurate and definitely more feasible. Consequently, four biological markers assessed by immunohistochemistry (IHC), i.e. estrogen receptor (ER), progesterone receptor (PgR), HER2 and the cell proliferation marker Ki67 can classify breast cancer in five subtypes: luminal A (ER-positive and/or PgR-positive, HER2-negative and Ki67^low^), luminal B (ER-positive and/or PgR-positive, HER2-negative and Ki67^high^), luminal-HER2 (ER-positive and/or PgR-positive and HER2-positive), HER2-enriched (ER-negative, PgR-negative, HER2-positive) and triple-negative (ER-negative, PgR-negative, HER2-negative) [24]. Furthermore, with the use of two additional biological markers, cytokeratin 5 (CK5) and epidermal growth factor receptor (EGFR), triple-negative breast cancer (TNBC) tumors can be separated in two distinct subgroups, basal core phenotype (BCP) and non-basal core phenotype (non-BCP) triple-negative tumors.

Information regarding therapeutic response of breast cancer subtypes to dose-dense adjuvant chemotherapy is very limited. Therefore, in order to shed light on this issue we performed a pooled analysis of the two previously mentioned randomized HeCOG studies (HE10/97, HE10/00) [18], [20], [21] to evaluate the outcome of patients treated with dose-dense chemotherapy according to immunophenotypical subtypes. Individual patient data from the two studies were combined, since they were both dealing with the concepts of dose-dense and sequential chemotherapy administration, they used similar eligibility criteria for patient participation and shared an identical chemotherapy arm.

## Materials and Methods

### Clinical studies

The HE10/97 trial [18] was a randomized phase III trial in patients with high-risk node-negative or intermediate/high-risk node-positive operable breast cancer, comparing four cycles of epirubicin (E) followed by four cycles of intensified CMF (E-CMF) with three cycles of E, followed by three cycles of paclitaxel (T, Taxol®, Bristol Myers-Squibb, Princeton, NJ) followed by three cycles of intensified CMF (E-T-CMF). All cycles were given every two weeks with G-CSF support. Dose intensity of all drugs in both treatment arms was identical, but cumulative doses and duration of chemotherapy period differed. Totally, 595 eligible patients entered the study in a period of 3.5 years (1997–2000).

The HE10/00 trial [20], [21] was a randomized phase III trial, in which a total of 1,086 eligible patients with node-positive operable breast cancer were accrued in a period of 5 years (2000–2005). Patients were treated with E-T-CMF (exactly as in the HE10/97 trial) or with four cycles of epirubicin/paclitaxel (ET) combination (given on the same day) every three weeks followed by three cycles of intensified CMF every two weeks (ET-CMF). By study design, the cumulative doses and the duration of chemotherapy period were identical in the two arms but dose intensity of epirubicin and paclitaxel was double in the E-T-CMF arm.

Treatment schedules for the two studies are described in detail in Table S1. HER2-positive patients received trastuzumab upon relapse, as previously described [25]. Baseline characteristics and clinical outcomes of both trials have been published [18], [20], [21]. Primary tumor diameter, axillary nodal status and tumor grade were obtained from the pathology report. Clinical protocols were approved by local regulatory authorities and were also included in the Australian New Zealand Clinical Trials Registry (ANZCTR) and allocated the following Registration Numbers: ACTRN12611000506998 (HE10/97) and ACTRN12609001036202 (HE10/00). The present translational research protocol was approved by the Bioethics Committee of the Aristotle University of Thessaloniki School of Medicine under the general title “Molecular investigation of the predictive and/or prognostic role of important signal transduction pathways in breast cancer” (A7150/18-3-2008). All patients signed a study-specific written informed consent before randomization, which in addition to giving consent for the trial allowed the use of their biological material for future research purposes.

### Tissue microarray (TMA) construction

**Figure 1:**
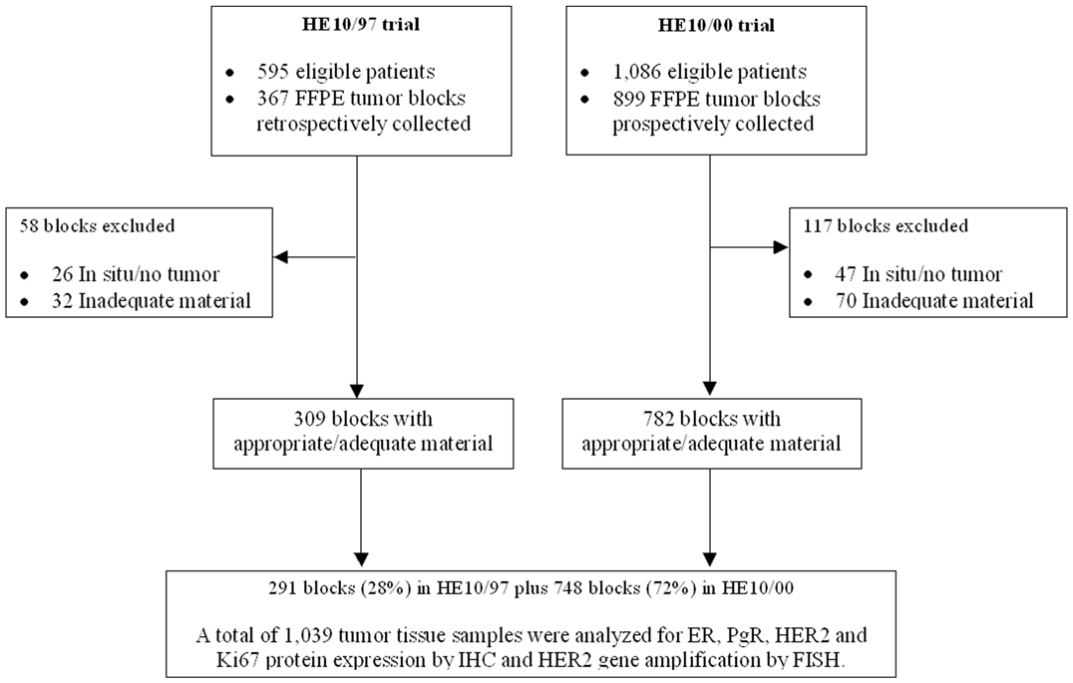
REMARK diagram.

FFPE tumor tissue samples (paraffin blocks) were collected retrospectively in the first trial (HE10/97) and prospectively in the second (HE10/00). The REMARK diagram [26] for the study is shown in Figure 1. Representative hematoxylin-eosin stained sections from the tissue blocks were reviewed by two experienced in breast cancer pathologists (M.B. and D.T.) and the most representative tumor areas were marked for the construction of the ΤΜΑ blocks with the use of a manual arrayer (Model I, Beecher Instruments, San Prairie, WI), as previously described [27], [28]. Each case was represented by 2 tissue cores, 1.5 mm in diameter, obtained from the most representative tumor areas of primary invasive or in some cases (9.6%) metastatic breast carcinomas and re-embedded in 51 microarray blocks. Each TMA block contained 38 to 66 tissue cores from the original tumor tissue blocks, while cores from various neoplastic, non-neoplastic and reactive tissues were also included, serving as assay controls. Cases not represented, damaged or inadequate on the TMA sections were re-cut from the original blocks and these sections were used for protein and gene analysis.

### Immunohistochemistry (IHC)

IHC for ER, PgR, HER2, Ki67, CK5 and EGFR was performed on serial 2.5 μm thick sections, using the Bond Max^TM^ and Bond III^TM^ autostainers (Leica Microsystems, Wetzlar, Germany), as previously described in detail [29]. The immunohistochemical target proteins, the source and dilution of antibodies used and the staining procedures for each target protein are presented in Table 1. To assure optimal immunoreactivity, the sections of the TMA blocks were stained in one run for each antibody, at the Laboratory of Molecular Oncology of the Hellenic Foundation for Cancer Research, Aristotle University of Thessaloniki School of Medicine. All cases were also stained for vimentin and cytokeratin 8/18, which were used as control stains for tissue immunoreactivity and fixation, as well as identification of tumor cells. Tissue samples negative for the above antibodies were excluded from the study. The evaluation of all IHC sections was done by experienced in breast cancer pathologists, blinded as to the patients' clinical characteristics and survival data.

**Table 1:** Proteins, source and dilution of antibodies, staining procedures and patterns and interpretation analysis.

Protein	Antibody clone, (source)	Antibody dilution	Antigen Retrieval/T	Pretreatment	Incubation Time	IHC Staining Detection System/Chromogen	Scoring System	Cut-off	Staining pattern	Ref.
*Intermediate* *filaments*										
CK5*	XM26 (1)	1∶50	HP/98°C	20′/ER2	20′	Polymer HRP + DAB	SQ	Any+	C	[25]
CK8/18	5D3 (1)	1∶100	HP/98°C	20′/ER1	20′	Polymer HRP + DAB	SQ	Any+	C	[25]
Vimentin*	V9 (2)	1∶500	HP/98°C	15′/ER1	20′	Polymer HRP + DAB	SQ	Any+	C	[25]
*Cell proliferation*										
Ki67*	MIB1 (2)	1∶70	HP/98°C	15′/ER2	20′	Polymer HRP + DAB	SQ	≥14%	N	[24]
*Cell receptors*										
EGFR	31G7 (3)	1∶50	HP/37°C	8′/ENZ	20′	Polymer HRP + DAB	0–3+^1^	≥1%	M	[27]
HER2	PL (2)	1∶200	HP/98°C	25′/ER1	30′	Polymer HRP + DAB	0–3+^2^	>30%	M	[28]
*Hormonal receptors*										
ER	6F11 (1)	1∶70	HP/98°C	20′/ER1	20′	Polymer HRP + DAB	H-Score	≥1%	N	[26]
PgR	1A6 (1)	1∶70	HP/98°C	20′/ER1	20′	Polymer HRP + DAB	H-Score	≥1%	N	[26]

DAB: 3,3′-Diaminobenzidine; C: Cytoplasmic; EGFR: epidermal growth factor receptor; ENZ: Proteolytic enzyme; ER1: Citric acid, pH 6.0;

ER2: ethylenediaminetetraacetate, pH 8.8; HP: Hot plate; HRP: Horseradish peroxidase; M: Membranous; N: Nuclear; SQ: semi-quantitative.

(1): Leica Biosystems, Newcastle, UK; (2): Dako, Glostrup, Denmark; (3): Invitrogen, Carlsbad, CA.

1 Scores 1+, 2+ and 3+ were considered to be positive; ^2^Score 3+ in >30% of tumor cells was considered to be positive.

* Double IHC was performed for the following antibodies: CK5/p53, CK14/Ki67 and CK17/Vimentin in the TMAs of the HE10/97 trial.

### Interpretation of the IHC results

ER, PgR, HER2, Ki67, CK5 and EGFR protein expression was evaluated in both tissue cores, according to the established or proposed criteria [30]–[34] presented in Table 1. The mean percentage of stained cells from the two cores was calculated by two independent pathologists. In cases with different intensities, the higher intensity score obtained from the two cores was used. If one of the tissue cores was lost or damaged the overall score was determined from the remaining one. When whole tissue sections were used, the entire tumor area was evaluated.

### Fluorescence in situ hybridization (FISH)

TMA sections or whole tissue sections (5 μm thick) were used for FISH analysis of HER2, using the ZytoLight^®^ SPEC HER2/TOP2A/CEN17 triple color probe (ZytoVision, Bremerhaven, Germany), as previously described [35]. Four carcinoma cell lines (MDA-MB-231, MDA-MB-175, MDA-MB-453, and SK-BR-3) from the Oracle HER2 Control Slide (Leica Biosystems), with a known HER2 gene status, were also used as a control of the FISH assays and analyzed for HER2 genomic status. TOP2A gene amplification was not evaluated for the purposes of the present study, since this marker is not used for breast cancer subtyping.

For the evaluation of the HER2 gene status, non-overlapping nuclei from the invasive part of the tumor were randomly selected and scored. The virtual slides of HER2, ER or PgR stains, created as previously described [36], were used for selecting the invasive part of the tumor in each TMA. Twenty tumor nuclei were counted according to Press et al [37]. The HER2 gene was considered to be amplified when the ratio of the gene probe/centromere probe was ≥2.2 [34], or the HER2 copy number was >6 [38]. In cases with values at or near the cut-off (1.8–2.2), additional 20 or 40 nuclei were counted and the ratio was recalculated. In cases with a borderline ratio at 60 nuclei, additional FISH assays were performed in whole sections. HER2 status was considered to be positive if HER2 was amplified (ratio ≥2.2 or copy number >6) by FISH and/or a HER2 score of 3+ was obtained by IHC.

### Statistical Analysis

Categorical data are presented as frequencies and corresponding percentages, while continuous data are presented as median and range values. The Fisher's exact or Pearson chi-square tests were used for group comparison of categorical data, while for continuous data the non-parametric Mann-Whitney or the Kruskall Wallis tests were used where appropriate. DFS was measured from the date of randomization until recurrence of tumor or secondary neoplasm or death from any cause [39]. OS was measured from the date of randomization until death from any cause. Surviving patients were censored at the date of last contact. Time from relapse to death was also estimated. Time-to-event distributions were presented using Kaplan-Meier curves.

Univariate Cox regression analyses were performed to assess the relationship of breast cancer subtype classification with OS or DFS. A backward selection procedure with a removal criterion of p>0.10 was performed in the multivariate Cox regression analysis in order to identify significant factors among age, paclitaxel treatment (yes vs. no), involved axillary lymph nodes (≥4 vs. 0–3), tumor grade (III-Undifferentiated vs. I–II), tumor size (>5 cm; 2–5 cm vs. ≤2 cm), type of surgery (yes vs. no), histology type (invasive lobular; mixed; other vs. invasive ductal), adjuvant hormonotherapy/radiotherapy (yes; missing vs. no) and breast cancer subtype classification (luminal B; luminal-HER2; HER2-enriched; TNBC vs. luminal A).

Results of this study were presented according to reporting recommendations for tumor marker prognostic studies [26]. In the present analysis, DFS and OS data were updated on March 2012. All statistical tests were two sided, and p<0.05 was considered statistically significant. The statistical analysis was conducted using SPSS (SPSS for Windows, version 15.0, SPSS Inc.).

## Results

A total of 1,039 patients with available FFPE tumor tissue blocks were included in the analysis. Selected patient and tumor characteristics according to treatment group (E-T-CMF vs E-CMF vs ET-CMF), paclitaxel or non-paclitaxel-containing regimens and clinical trial are depicted in Tables S2, S3 and S4, respectively. Of note, there were significant differences in a number of important tumor characteristics among patients with and without blocks available. Patients with available blocks presented with higher frequency of ≥4 positive nodes (both trials) and tumor size >2 cm, adjuvant RT and grade 3 tumors (HE10/00 trial), which might have contributed to an extent to the increased availability of tissue blocks for research purposes in these patients (Table S5). Importantly, no differences were detected for DFS and OS between patients with or without available blocks.

Patients were classified, according to the 6 biological markers assessed, as luminal A (ER-positive and/or PgR-positive, HER2-negative and Ki67^low^), luminal B (ER-positive and/or PgR-positive, HER2-negative and Ki67^high^), luminal-HER2 (ER-positive and/or PgR-positive and HER2-positive), HER2-enriched (ER-negative, PgR-negative, HER2-positive), TNBC (ER-negative, PgR-negative, HER2-negative) and BCP (TNBC, CK5-positive and/or EGFR-positive). Basic clinico-pathological variables varied by breast cancer immunophenotypical sybtypes as shown in Table 2. Grade 3 tumors comprised only about a quarter of luminal A tumors, half of luminal B, and the majority of luminal-HER2, HER2-enriched, TNBC and BCP tumors (56.3%, 76.4%, 69.2% and 75.2%, respectively). Invasive ductal histology was found in over 80% of luminal-HER2, HER2-enriched, TNBC and BCP tumors (85.9%, 88.2%, 80.5% and 81.2%, respectively). In this cohort with worse pathological characteristics among the high-risk patients entering an adjuvant chemotherapy trial, the age distribution differed between subtypes. Tumors of the luminal-HER2 subtype were more often encountered in younger (less than 50 year-old) patients, followed by luminal B and TNBC. Modified radical mastectomy had been performed more frequently in patients with HER2-enriched tumors, while adjuvant hormonal therapy was given more often in patients with luminal tumors, as would be expected. Of note, in the present analysis the subtypes were defined following central assessment, while patients in both trials were treated according to the evaluation of the ER and PgR status at the local laboratories. The observed agreement for ER, PgR and HER2 was 82.6%, 76.1% and 84.3%, respectively, with a kappa of 58.1%, 48.0% and 61.6%, respectively.

**Table 2:** Selected patient and tumor characteristics according to breast cancer subtypes defined by immunohistochemistry.

		Luminal A	Luminal B	Luminal-HER2	HER2-enriched	TNBC	*BCP* *
		N = 258	N = 396	N = 142	N = 110	N = 133	*N = 101*
Age in years^1^	Median (range)	55 (22–79)	52 (25–78)	49 (24–79)	54 (25–77)	53 (22–75)	*53 (22–72)*
N of positive nodes^2^	Median (range)	4 (0–32)	5 (0–43)	5 (0–35)	5 (1–35)	4 (0–54)	*4 (0–54)*

		N (%)	N (%)	N (%)	N (%)	N (%)	*N (%)*
Study^3^	HE1097	34 (13.2)	144 (36.4)	42 (29.6)	32 (29.1)	39 (29.3)	*31 (30.7)*
	HE1000	224 (86.8)	252 (63.6)	100 (70.4)	78 (70.9)	94 (70.7)	*70 (69.3)*
Treatment^4^	Non-paclitaxel	16 (6.2)	87 (22.0)	21 (14.8)	13 (11.8)	19 (14.3)	*12 (11.9)*
	Paclitaxel	242 (93.8)	309 (78.0)	121 (85.2)	97 (88.2)	114 (85.7)	*89 (88.1)*
Age	<34	6 (2.3)	20 (5.1)	4 (2.8)	4 (3.6)	6 (4.5)	*6 (5.9)*
	34–50	96 (37.2)	147 (37.1)	70 (49.3)	35 (31.8)	52 (39.1)	*41 (40.6)*
	>50	156 (60.2)	229 (57.8)	68 (47.9)	71 (64.5)	75 (56.4)	*54 (53.5)*
Menopausal status	Premenopausal	112 (43.4)	191 (48.2)	74 (52.1)	47 (42.7)	59 (44.4)	*47 (46.5)*
	Postmenopausal	146 (56.6)	205 (51.8)	68 (47.9)	63 (57.3)	74 (55.6)	*54 (53.5)*
Type of surgery^5^	MRM	179 (69.4)	277 (69.9)	95 (66.9)	84 (76.4)	77 (57.9)	*49 (48.5)*
	Breast conserving	79 (30.6)	119 (30.1)	47 (33.1)	26 (23.6)	56 (42.1)	*52 (51.5)*
Tumor size (cm)	≤2	89 (34.5)	121 (30.6)	40 (28.2)	26 (23.6)	42 (31.6)	*37 (36.6)*
	2–5	140 (54.3)	224 (56.6)	84 (59.2)	70 (63.6)	75 (56.4)	*54 (53.5)*
	>5	29 (11.2)	51 (12.9)	18 (12.7)	14 (12.7)	16 (12.0)	*10 (9.9)*
N of positive nodes^6^	0	-	1 (0.3)	2 (1.4)	-	1 (0.8)	*1 (1.0)*
	1–4	122 (47.3)	143 (36.1)	50 (35.2)	37 (33.6)	49 (36.8)	*39 (38.6)*
	≥4	136 (52.7)	252 (63.6)	90 (63.4)	73 (66.4)	83 (62.4)	*61 (60.4)*
Histological grade^7^	1	30 (11.6)	13 (3.3)	4 (2.8)	2 (1.8)	3 (2.3)	*3 (3.0)*
	2	164 (63.6)	183 (46.2)	57 (40.1)	24 (21.8)	38 (28.6)	*22 (21.8)*
	3	64 (24.8)	199 (50.3)	80 (56.3)	84 (76.4)	92 (69.2)	*76 (75.2)*
	Undifferentiated	-	1 (0.3)	1 (0.7)	-	-	*-*
Histology type^8^	Invasive ductal	184 (71.3)	294 (74.2)	122 (85.9)	97 (88.2)	107 (80.5)	*82 (81.2)*
	Invasive lobular	41 (15.9)	45 (11.4)	8 (5.6)	2 (1.8)	10 (7.5)	*4 (4.0)*
	Mixed	22 (8.5)	39 (9.8)	7 (4.9)	6 (5.5)	2 (1.5)	*2 (2.0)*
	Other	11 (4.3)	18 (4.5)	5 (3.5)	5 (4.5)	14 (10.5)	*13 (12.9)*
Adjuvant HT^9^		226 (87.6)	344 (86.9)	120 (84.5)	52 (47.3)	63 (47.4)	*50 (49.5)*
Adjuvant RT		184 (71.3)	300 (75.8)	112 (78.9)	86 (78.2)	103 (77.4)	*81 (80.2)*

* BCP  = 75.9% of TNBC.

BCP, basal core phenotype; HT, hormonal therapy; MRM, modified radical mastectomy; N, number; RT, radiotherapy; TNBC, triple-negative breast cancer.

1 p = 0.004, ^2^p = 0.013, ^3^p<0.001, ^4^p<0.001,^ 5^p = 0.030, ^6^p = 0.031,^ 7^p<0.001, ^8^p<0.001, ^9^p<0.001/

Patients were classified as: luminal A (ER*-positive* and/or PgR-positive, *HER2-negative, Ki67^low^*); luminal B (ER*-positive* and/or PgR-positive, *HER2-negative, Ki67^high^*); luminal-HER2 (ER*-positive* and/or PgR-positive, HER2-positive); HER2-enriched (ER-negative, PgR-negative, HER2-positive); triple-negative (TNBC) (ER-negative, PgR-negative, HER2-negative); and basal core phenotype (BCP) (TNBC, CK5-positive and/or EGFR-positive).

After a median follow-up time of 105.4 months (range, 0.1–166.7), 5-year and 10-year DFS rates for all patients in the study were 73.1% and 60.3%, respectively. Likewise, 5-year and 10-year OS rates were 86.1% and 70.6%, respectively. (Table S6). Survival status and site of first relapse according to breast cancer subtype is shown in Table 3. Locoregional relapses occurred more frequently in patients with TNBC, while distant relapses were more rare in patients with luminal A tumors compared to the other subtypes. Notably, liver metastases were most often recorded in patients with HER2-enriched tumors, while brain tumors in patients with TNBC. Bone was the most frequent site of metastasis in patients with luminal-HER2 and luminal B tumors. Finally, both locoregional and distant metastases were seen far less in patients with luminal A tumors.

**Table 3:** Survival status according to breast cancer subtypes defined by immunohistochemistry (for subtype description see Table 2 legend).

	Luminal A (N = 258)	Luminal B (N = 396)	Luminal-HER2 (N = 142)	HER2-enriched (N = 110)	TNBC (N = 133)	*BCP* * * (N = 101)*
**Disease-free survival**						
Progressions N (%)	48 (18.6)	122 (30.8)	52 (36.6)	39 (35.5)	50 (37.6)	*35 (34.7)*
Events N (%)	66 (25.6)	152 (38.4)	60 (42.3)	40 (36.4)	59 (44.4)	*40 (39.6)*
5-year rate (%)	83.5	75.7	64.0	67.0	60.6	*64.4*
10-year rate (%)	66.6	58.1	56.1	63.2	56.2	*59.5*
Range	6.0–121.5	4.0–160.5	5.3–109.3	5.1–77.0	6.9–143.0	*9.6–89.9*
**Overall survival**						
Deaths N (%)	39 (15.1)	111 (28.0)	46 (32.4)	27 (24.5)	52 (39.1)	*35 (34.7)*
5-year rate (%)	92.1%	88.2%	85.8%	83.3%	70.5%	*73.3*
10-year rate (%)	79.4%	69.8%	62.6%	72.1%	61.7%	*65.6*
Range	25.7–143.4	4.3–160.5	10.5–120.6	13.0–110.9	8.1–151.3	*13.6–151.3*
**Relapses N (%)**						
Locoregional relapse^1^	6 (2.3)	16 (4.0)	7 (4.9)	9 (8.2)	14 (10.5)	*12 (11.9)*
Distant relapse^2^	44 (17.1)	114 (28.8)	42 (29.6)	35 (31.8)	41 (30.8)	*26 (25.7)*
Brain^3^	2 (0.8)	8 (2.0)	2 (1.4)	1 (0.9)	8 (6.0)	*6 (5.9)*
Lung	15 (5.8)	34 (8.6)	13 (9.2)	14 (12.7)	15 (11.3)	*9 (8.9)*
Liver^4^	12 (4.7)	33 (8.3)	11 (7.7)	18 (16.4)	7 (5.3)	*3 (3.0)*
Bones^5^	21 (8.1)	60 (15.2)	22 (15.5)	9 (8.2)	14 (10.5)	*7 (6.9)*
Soft tissue/nodes	5 (1.9)	16 (4.0)	5 (3.5)	6 (5.5)	7 (5.3)	*7 (6.9)*
Visceral^6^	26 (10.1)	66 (16.7)	25 (17.6)	29 (26.4)	28 (21.1)	*18 (17.8)*
Locoregional & distant	3 (1.2)	8 (2.0)	1 (0.7)	5 (4.5)	4 (3.0)	*3 (3.0)*

* BCP  = 75.9% of TNBC.

BCP, basal core phenotype; N, number; TNBC, triple-negative breast cancer.

1 p = 0.005; ^2^p = 0.003; ^3^p = 0.023; ^4^p = 0.006; ^5^p = 0.03; ^6^p = 0.001.

There were no significant differences in DFS or OS among patients treated with the three different regimens (Figure S1). The subtype classification was prognostic for both DFS and OS. Setting luminal A as the referent category, we observed an increased risk for relapse and death in all subtypes. The hazard ratio (HR), 95% confidence interval (CI) and p value for each subtype for DFS and OS are shown in Table 4. Within the TNBC subtype, BCP tumors were associated with lower risk for relapse compared to non-BCP tumors (Figure S2), however the number of patients in the two subgroups is small for meaningful comparisons.

**Figure 2:**
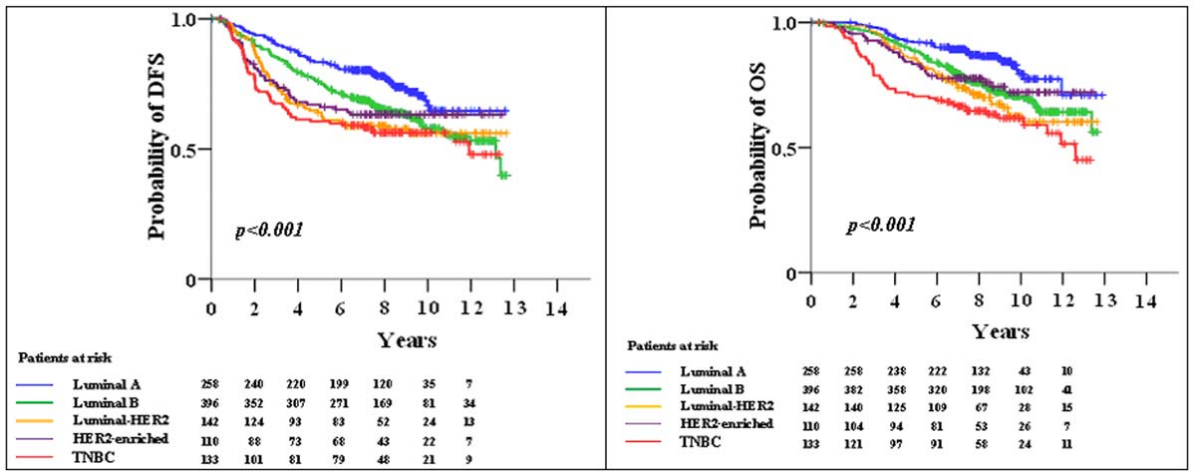
DFS and OS according to breast cancer subtypes. Patients were classified as: luminal A (ER-positive and/or PgR-positive, HER2-negative, Ki67^low^); luminal B (ER-positive and/or PgR-positive, HER2-negative, Ki67^high^); luminal-HER2 (ER-positive and/or PgR-positive, HER2-positive); HER2-enriched (ER-negative, PgR-negative, HER2-positive); and triple-negative (TNBC) (ER-negative, PgR-negative, HER2-negative).

As presented in the survival plots (Figure 2), patients with TNBC, luminal-HER2 and HER2-enriched tumors had, in the pre-trastuzumab era for early disease, a high risk for relapse in the first 5 to 6 years from initial diagnosis, while patients with luminal A and B tumors had a lower but almost constant risk for relapse across all years. Concerning OS, again patients with TNBC had an increased risk for death in the first 4 to 5 years, while patients with luminal-HER2 and HER2-enriched tumors, despite similar relapse rates, showed a much lower risk for death compared to TNBC patients, probably reflecting the beneficial effect of trastuzumab given at relapse in most cases [25].

**Table 4:** Univariate Cox regression analysis for breast cancer subtypes (for subtype description see Table 2 legend).

	DFS			OS		
	Hazard Ratio	95% CI	Wald's p value	Hazard Ratio	95% CI	Wald's p value
Luminal B vs. Luminal A	1.54	1.15–2.06	0.003	1.83	1.27–2.63	0.001
Luminal-HER2 vs. Luminal A	1.87	1.32–2.65	<0.001	2.21	1.44–3.39	<0.001
HER2-enriched vs. Luminal A	1.61	1.07–2.38	0.018	1.70	1.04–2.78	0.034
TNBC vs. Luminal A	2.10	1.48–2.98	<0.001	3.05	2.02–4.63	<0.001

DFS, disease-free survival; OS, overall survival; CI, confidence interval; TNBC, triple-negative breast cancer.

**Figure 3:**
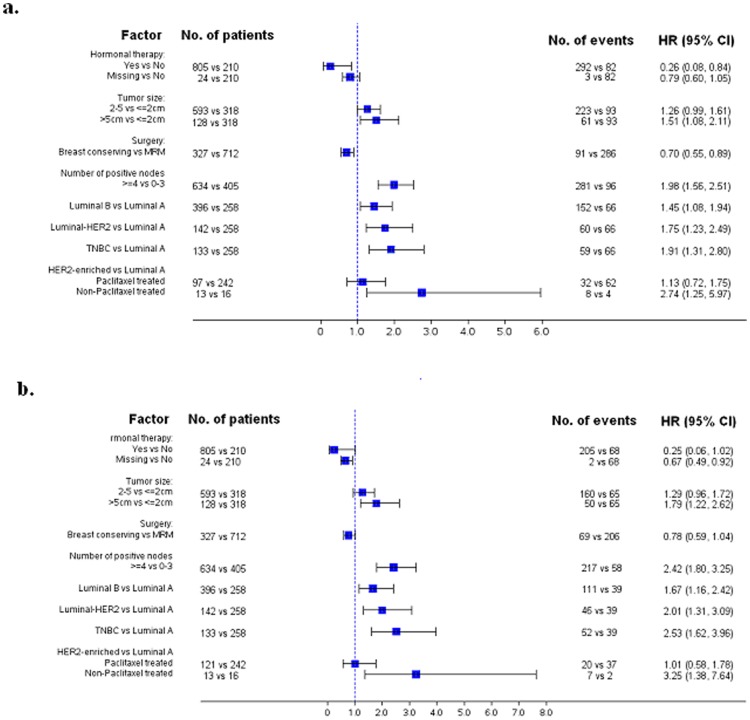
Forest plots from multivariate Cox regression models: DFS (a) and OS (b) (for subtype description see **Figure 2** legend).

Among patients with relapse (n = 311), 70.4% died. The median time from relapse to death was significantly shorter for TNBC patients (median = 16.7, 95% CI: 12.3–21.1 months), compared to the luminal A (median = 44.8, 95% CI: 23.1–66.5 months, p<0.001), luminal B (median = 33.2, 95% CI: 28.3–38.1 months, p = 0.006), luminal-HER2 (median = 43.0, 95% CI: 30.3–55.7 months, p = 0.006) and HER2-enriched (median = 35.4, 95% CI: 28.1–42.8 months, p = 0.011). When adjusting for treatment and other prognostic factors, all subtypes had worse DFS and OS than the luminal A subtype (Figure 3).

**Figure 4:**
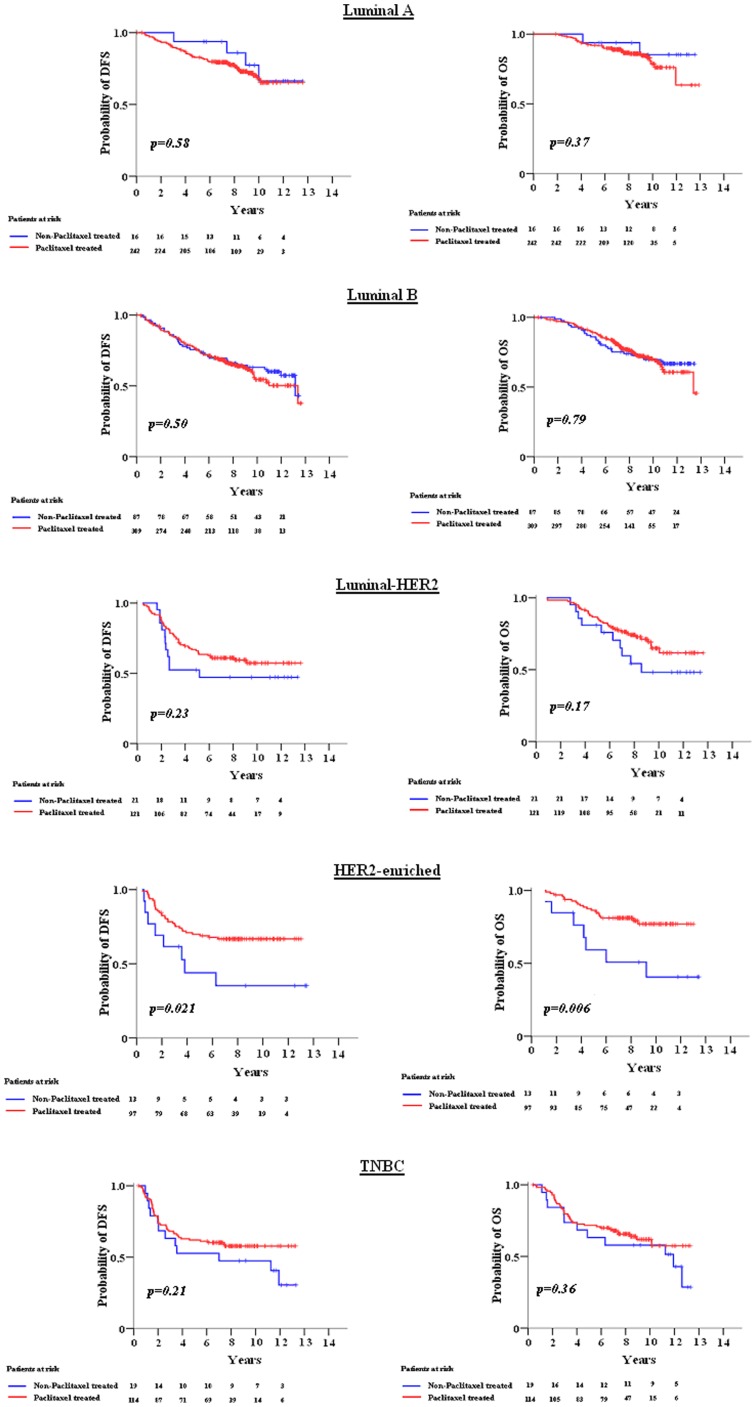
DFS and OS in paclitaxel and non-paclitaxel treated patients according to breast cancer subtypes (for subtype description see **Figure 2** legend).

DFS and OS were compared in a predefined analysis between taxane containing treatment arms (E-T-CMF and ET-CMF) and the non-taxane containing treatment (E-CMF), in each IHC-defined subtype group. A significant benefit from paclitaxel containing regimens for both DFS and OS (log-rank, p = 0.021 and p = 0.006, respectively) was noticed among patients with HER2-enriched tumors (Figure 4).

In the multivariate analysis (Figure 3), apart from any subtype compared to luminal A, higher tumor size, and ≥4 positive nodes were adverse prognostic factors for both DFS and OS, with modified radical mastectomy (MRM) surgery being an adverse prognostic factor for DFS and absence of hormonal therapy for OS. Adjusting for the prognostic factors, a significant benefit from the taxane containing treatment was found in the HER2-enriched subtype, with estimated HR = 0.44 (95% CI: 0.20–0.98; interaction p = 0.037) and HR = 0.32 (95% CI: 0.13–0.78; interaction p = 0.014), for DFS and OS, respectively. Treatment with taxanes was not associated with DFS or OS in the luminal A, luminal B, luminal-HER2 and TNBC subtypes.

## Discussion

In the present study we investigated the clinical outcome of patients with operable breast cancer classified to five well-established immunophenotypical breast cancer subtypes. These patients were included in two consecutively conducted adjuvant phase III trials with dose-dense sequential chemotherapy [18], [20], [21]. The five subtypes were defined with the use of a four-marker panel (ER, PgR, HER2 and Ki67), as also reported by others [24]. Notably, with the use of two additional biological markers, CK5 and EGFR we further separated triple negative tumors into two distinct subgroups, BCP and non-BCP.

The largest group of patients in our study was of the luminal B subtype (38.1%) followed by luminal A (24.8%). In contrast to the corresponding rate in large registry data sets, hospital-based or population-based case series [40]–[42], in which luminal B tumors represented only a small minority, about half of the tumors in our study were classified as luminal B/luminal-HER2. This is in keeping with the existing literature, for an “intermediate or high-risk” population entering a randomized trial with adjuvant chemotherapy in operable breast cancer [43], [44]. Conceivably, patients with more favorable prognosis, like those with luminal A tumors, are usually treated with less aggressive chemotherapeutic regimens followed by hormonal therapy or even with hormonal therapy alone.

Our study has several strengths, as well as limitations. It has been postulated that breast cancer subtypes are predictive and behave differently to specific treatments [40]. If so, response evaluation of these subtypes to modern chemotherapeutic regimens, in the context of randomized trials, is of great importance. In our study, tissue blocks were obtained from patients participating in two such trials, investigating the role of epirubicin, paclitaxel and CMF-containing dose-dense sequential chemotherapy, which shared similar eligibility criteria. Furthermore, all IHC and in situ hybridization testing was carried out in a central “high-volume” laboratory, negating inter-laboratory assay result variability and, in parallel, scoring was done by experienced pathologists. Therefore, the possibility for misclassification of tumors was significantly reduced.

On the other hand, tumor tissue blocks were retrospectively collected in the HE10/97 trial and prospectively in the HE10/00 trial. Therefore, in the present analysis, patients with available blocks may not be representative of the entire patient population enrolled in the two trials, since there were differences in tumor size, number of metastatic lymph nodes and tumor grade, which may lead to biases. Nevertheless, these variables were taken into account in the multivariate analyses. Furthermore, the size of both trials is considered to be intermediate according to current standards, which may preclude the identification of subtle but still biologically meaningful differences in prognosis, especially in subtypes with relatively small number of patients, i.e. BCP or non-BCP TNBC subtypes, if examined separately.

Since a considerable number of patients in the present analysis were not treated with paclitaxel, while all received epirubicin and CMF, we sought for potentially differential responses of the different subtypes to this agent. Eventually, we were able to demonstrate a particular benefit from the use of paclitaxel only in patients with HER2-enriched tumors. In a previously published study on FFPE tissue samples from 394 patients randomized in the HE10/97 trial, an association of HER2 status and outcome was not detected, maybe due to the smaller sample size and HER2 status classification based mainly on IHC [45]. However, in a large retrospective analysis of 1,322 FFPE tissue blocks from 3,121 women participating in the Cancer and Leukemia Group B 93441/INT0148 trial (four cycles of doxorubicin and cyclophosphamide followed by four cycles of paclitaxel or observation), Hayes et al [46] reported that a survival benefit from the addition of paclitaxel was evident in patients with HER2-positive tumors regardless of ER status.

Regarding the behavior of TNBC patients in response to treatment with taxanes, results in the literature are equivocal at best. An analysis of the Groupo Espanol de Investigacion en Cancer de Mama (GEICAM) 9906 trial has shown a superiority of the fluorouracil, epirubicin, cyclophosphamide (FEC) combination followed by weekly paclitaxel over FEC alone, which was more prominent in patients with TNBC [44]. In line with these data, Iwata et al [47] reported that patients with TNBC were more likely to achieve pathological complete response (pCR) to neoadjuvant chemotherapy with four cycles of docetaxel followed by four cycles of FEC compared to those with other subtypes. Conversely, in the Breast Cancer International Research Group (BCIRG)-001 trial the combination of docetaxel (Taxotere®), doxorubicin (Adriamycin®) and cyclophosphamide (TAC) offered a significant improvement in 3-year DFS in patients with luminal B (defined as ER-positive, PgR-positive and either Ki67^high^ or HER-positive), but only a marginal trend in patients with TNBC or HER2-enriched tumors over treatment with fluorouracil, doxorubicin, cyclophosphamide (FAC). Intriguingly, among patients with node-positive operable breast cancer randomized in the PACS01 trial comparing six cycles of adjuvant FEC with three cycles of FEC followed by three cycles of docetaxel [48], those with luminal A did not benefit from docetaxel. Conversely, patients with luminal B, HER2-overexpressing and triple negative tumors had a significant reduction in their risk for relapse. Ki67 positivity was the most powerful predictor of benefit from docetaxel [49]. We were not able to show a paclitaxel benefit in patients with TNBC. Whether this is due to differences in sample size, regimens used, patient populations or other unidentified factors cannot be revealed by the present analysis.

It has been shown that the pattern of first relapse varies among patients with different breast cancer subtypes [50]. That was also the case for the randomized patients analyzed in our study, in which locoregional relapses as well distant relapses were more frequently seen in patients with TNBC and HER2-enriched tumors, a finding that has been also observed by others in hospital-based case series [24], [51]. The 6% rate of brain metastases, recorded in our patients with TNBC at the 5-year time point, was almost identical to that reported in other large studies [51]–[53]. It is worth mentioning that HER2 is considered to be a robust risk factor for the development of CNS disease [54], [55]. A retrospective analysis of 9,524 patients enrolled in 10 adjuvant trials, led by the International Breast Cancer Study Group in the pre-trastuzumab era, reported that the 10-year cumulative incidence of brain as primary site of metastasis was 2.7% in patients with HER2-positive tumors and 1.0% in patients with HER2-negative tumors (p<0.01) [56]. The respective rate in our patients with HER2-positive disease was slightly above 1%. Finally, the present analysis confirmed the significant liver predisposition of metastases associated with HER2-enriched tumors reported by others [24], [41].

Notable differences in the behavior of breast cancer subtypes are not seen only in the pattern of metastases, but also in the time to first relapse. Data from 498 patients, enrolled in a randomized trial exploring the beneficial role of local cavity boost of RT to breast conserving therapy, indicated that median time to first event was significantly shorter for TNBC and HER2-enriched subtypes. Crude recurrence rates of luminal A tumors were less than one third of those seen in BCP tumors, regarding loco-regional relapses, and less than half regarding the development of distant metastases at 5 years [50]. In the study by Blows [40], which pooled individual data from more than 10,000 patients with invasive breast cancer from 12 studies with known status of ER, HER2 and at least one basal marker (CK5/6 or EGFR), it was clearly shown that even though for HER2-positive and non-luminal subtypes mortality rates tended to peak within 5 years from diagnosis, with longer follow-up the prognosis became poorer in the luminal subtypes. Unfortunately, in that study, information on Ki67 status was not available and this might have led to misclassification of patients with luminal B as luminal A. These data fit perfectly with the cumulative hazard plots extracted from the present analysis, with patients with TNBC and HER2-positive tumors having an increased risk for relapse and death in the first 5 to 6 years from initial diagnosis. On the contrary, patients with luminal tumors had a constant risk for relapse and death across time. Further, at 5 years, the DFS and OS rates for patients with luminal A tumors were 83.5% and 92.1%, while for those with TNBC were 60.6% and 70.5%, respectively.

In summary, the present pooled analysis of individual data of more than 1,000 patients with “intermediate or high-risk” operable breast cancer, according to the immunophenotypical subtypes of their tumor, indicated that in the presence of dose-dense chemotherapy, patients with TNBC have the worse prognosis and luminal A the best. For patients with HER2-positive disease, a similar pattern emerges up to recurrence, in the adjuvant pre-trastuzumab era, while prolongation of time from recurrence to survival is noted, probably because of the use of trastuzumab. A predictive role of the subtypes for benefit from paclitaxel was not identified, except for patients with HER2-enriched tumors. Clearly, more data, similar to those reported here, from other randomized trials and eventually a meta-analysis, would be needed to delineate the predictive value of each subtype and especially of TNBC, for which a complementary targeted treatment to dose-dense sequential chemotherapy does not presently exist.

## Supporting Information

Figure S1 **DFS and OS according to treatment regimens.**
(TIF)

Figure S2 **DFS and OS in patients with BCP and non-BCP tumors.**
(TIF)

Table S1 Treatment details for the HE10/97 and HE10/00 trials.
(DOC)

Table S2 Selected patient and tumor characteristics according to treatment group (for subtype description see Table 2 legend in manuscript).
(DOC)

Table S3 Selected patient and tumor characteristics according to paclitaxel or non-paclitaxel containing regimens (for subtype description see Table 2 legend in manuscript).
(DOC)

Table S4 Selected patient and tumor characteristics according to clinical trial (for subtype description see Table 2 legend in manuscript).
(DOC)

Table S5 Comparison of patients with and without available blocks in the two trials.
(DOC)

Table S6 Survival status for all patients.
(DOC)
