# Supplementary figures and images for: Differential Response of Immunohistochemically Defined Breast Cancer Subtypes to Anthracycline-Based Adjuvant Chemotherapy with or without Paclitaxel
Source: PLoS One. 2012 Jun 5;7(6):e37946. doi: 10.1371/journal.pone.0037946 (PMC3367950; doi:10.1371/journal.pone.0037946)

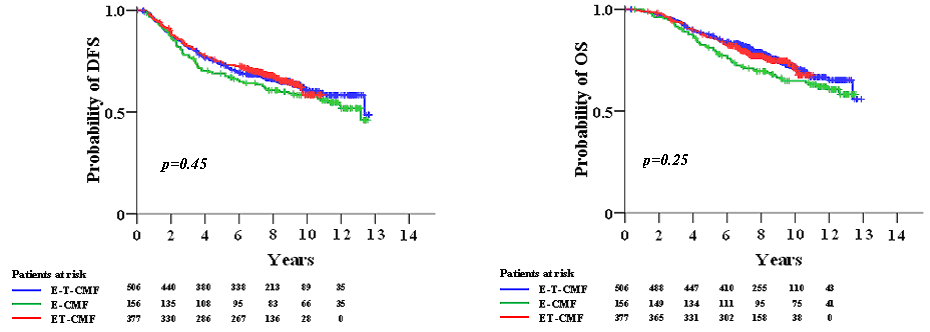

Supplement: Figure S1 — DFS and OS according to treatment regimens. (TIF) [file pone.0037946.s001.tif]

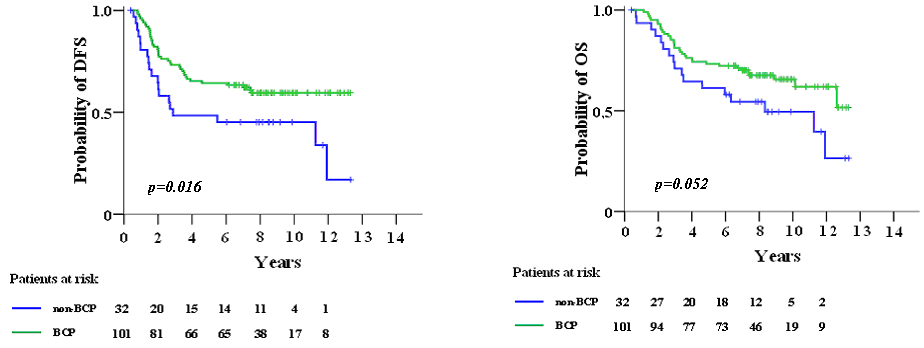

Supplement: Figure S2 — DFS and OS in patients with BCP and non-BCP tumors. (TIF) [file pone.0037946.s002.tif]
