# Supplementary material for: Differential Response of Immunohistochemically Defined Breast Cancer Subtypes to Anthracycline-Based Adjuvant Chemotherapy with or without Paclitaxel
Source: PLoS One. 2012 Jun 5;7(6):e37946. doi: 10.1371/journal.pone.0037946 (PMC3367950; doi:10.1371/journal.pone.0037946)
Supplement: Table S1 — Treatment details for the HE10/97 and HE10/00 trials. (DOC) [file pone.0037946.s003.doc]

| **Trial** | **N** | **n** | **Treatment schedule** |
| --- | --- | --- | --- |
| HE10/97 | **595** | 309 | **E-T-CMF:** Epirubicin 110 mg/m^2^ q 2 weeks x 3 followed by Paclitaxel 250 mg/m^2^ q 2 weeks x 3 followed by Cyclophosphamide 840 mg/m^2^; methotrexate 57 mg/m^2^; fluoroucacil 840 mg/m^2^ (CMF) q 2 weeks x 3. GCSF support in all cycles. ***vs.*** **E-CMF:** Epirubicin 110 mg/m^2^ q 2 weeks x 4 followed by Cyclophosphamide 840 mg/m^2^; methotrexate 57 mg/m^2^; fluoroucacil 840 mg/m^2^ (CMF) q 2 weeks x 4. GCSF support in all cycles. Patients with ER/PgR-positive tumors received tamoxifen 20 mg daily for five years. Premenopausal patients received additional treatment with an LH-RH analog for two years. All patients who underwent partial mastectomy or with tumors >5 cm and/or with ≥4 infiltrated axillary nodes, irrespectively of the type of surgery, were irradiated. Radiation therapy and hormonal therapy were administered after the completion of chemotherapy. |
| HE10/00 | **1,086** | 782 | **E-T-CMF:** As in HE10/97 trial. ***vs.*** **ET-CMF:** Epirubicin 83 mg/m^2^ + Paclitaxel 187 mg/m^2^ q 3 weeks x 4 followed by Cyclophosphamide 840 mg/m^2^; methotrexate 57 mg/m^2^; fluoroucacil 840 mg/m^2^ (CMF) q 2 weeks x 3. GCSF support in all cycles with CMF. Premenopausal patients received hormonal therapy as in the HE10/97 trial. Postmenopausal patients received tamoxifen 20 mg daily for 2-3 years followed 2-3 years of daily examestane 25 mg. Criteria for irradiation were the same as in the HE10/97 trial. |

N: number of eligible patients; n: number of FFPE tissue blocks.
