# Supplementary material for: Differential Response of Immunohistochemically Defined Breast Cancer Subtypes to Anthracycline-Based Adjuvant Chemotherapy with or without Paclitaxel
Source: PLoS One. 2012 Jun 5;7(6):e37946. doi: 10.1371/journal.pone.0037946 (PMC3367950; doi:10.1371/journal.pone.0037946)
Supplement: Table S2 — Selected patient and tumor characteristics according to treatment group (for subtype description see Table 2 legend in manuscript). (DOC) [file pone.0037946.s004.doc]

|  | | **E-T-CMF** | E-CMF | **ET-CMF** | **All patients** |
| --- | --- | --- | --- | --- | --- |
|  | | **N=506** | **N=156** | **N=377** | **N=1,039** |
| Age in years | Median (range) | 52 (24-79) | 51 (22-78) | 54 (22-76) | 53 (22-79) |
| N of positive nodes^1^ | Median (range) | 5 (0-54) | 6 (0-35) | 4 (0-40) | 4 (0-54) |
|  |  | **N (%)** | **N (%)** | **N (%)** | **N (%)** |
| Age | <34 | 17 (3.4) | 6 (3.8) | 17 (4.5) | 40 (3.8) |
|  | 34-50 | 208 (41.1) | 68 (43.6) | 124 (32.9) | 400 (38.5) |
|  | >50 | 281 (55.5) | 82 (52.6) | 236 (62.6) | 599 (57.7) |
| Menopausal status | Premenopausal | 239 (47.2) | 81 (51.9) | 163 (43.2) | 483 (46.5) |
|  | Postmenopausal | 267 (52.8) | 75 (48.1) | 214 (56.8) | 556 (53.5) |
| Type of surgery^2^ | MRM | 350 (69.2) | 119 (76.3) | 243 (64.5) | 712 (68.5) |
|  | Breast conserving | 156 (30.8) | 37 (23.7) | 134 (35.5) | 327 (31.5) |
| Tumor size (cm)^3^ | <2 | 145 (28.7) | 57 (36.5) | 116 (30.8) | 318 (30.6) |
|  | 2-5 | 302 (59.7) | 71 (45.5) | 220 (58.4) | 593 (57.1) |
|  | >5 | 59 (11.7) | 28 (17.9) | 41 (10.9) | 128 (12.3) |
| Histological type | Invasive ductal | 401 (79.2) | 112 (71.8) | 291 (77.2) | 804 (77.4) |
|  | Invasive lobular | 42 (8.3) | 19 (12.2) | 45 (11.9) | 106 (10.2) |
|  | Mixed | 39 (7.7) | 16 (10.3) | 21 (5.6) | 76 (7.3) |
|  | Other | 24 (4.7) | 9 (5.8) | 20 (5.3) | 53 (5.1) |
| Number of positive nodes^4^ | 0 | 2 (0.4) | 2 (1.3) | - | 4 (0.4) |
|  | 1-4 | 183 (36.2) | 39 (25.0) | 179 (47.5) | 401 (38.6) |
|  | ≥4 | 321 (63.4) | 115 (73.7) | 198 (52.5) | 634 (61.0) |
| Adjuvant HT^5^ |  | 384 (75.9) | 137 (87.8) | 284 (75.3) | 805 (77.5) |
| Adjuvant RT |  | 373 (73.7) | 123 (78.8) | 289 (76.7) | 785 (75.6) |
| Histological grade^6^ | 1 | 26 (5.1) | 4 (2.6) | 22 (5.8) | 52 (5.0) |
|  | 2 | 207 (40.9) | 88 (56.4) | 171 (45.4) | 466 (44.9) |
|  | 3 | 271 (53.6) | 64 (41.0) | 184 (48.8) | 519 (50.0) |
|  | Undifferentiated | 2 (0.4) | - | - | 2 (0.2) |
| Ki67^7^ | <14 | 166 (32.86) | 27 (17.3) | 147 (39.0) | 340 (32.7) |
|  | ≥14 | 335 (66.2) | 129 (82.7) | 228 (60.5) | 692 (66.6) |
|  | Missing data | 5 (1.0) | - | 2 (0.5) | 7 (0.7) |
| ER | Negative | 152 (30.0) | 42 (26.9) | 87 (23.1) | 281 (27.0) |
|  | Positive | 353 (69.8) | 113 (72.4) | 288 (76.4) | 754 (72.6) |
|  | Missing data | 1 (0.2) | 1 (0.6) | 2 (0.5) | 4 (0.4) |
| PgR | Negative | 174 (34.4) | 54 (34.6) | 115 (30.5) | 343 (33.0) |
|  | Positive | 331 (65.4) | 102 (65.4) | 262 (69.5) | 695 (66.9) |
|  | Missing data | 1 (0.2) | - | - | 1 (0.1) |
| HER2 status | Negative | 376 (74.3) | 122 (78.2) | 289 (76.7) | 787 (75.7) |
|  | Positive | 130 (25.7) | 34 (21.8) | 88 (23.3) | 252 (24.3) |
| Tumor subtype^8^ | Luminal A | 127 (25.1) | 16 (10.3) | 115 (30.5) | 258 (24.8) |
|  | Luminal B | 181 (35.8) | 87 (55.8) | 128 (34.0) | 396 (38.1) |
|  | Luminal-HER2 | 66 (13.0) | 21 (13.5) | 55 (14.6) | 142 (13.7) |
|  | HER2-enriched | 64 (12.6) | 13 (8.3) | 33 (8.8) | 110 (10.6) |
|  | TNBC | 68 (13.4) | 19 (12.2) | 46 (12.2) | 133 (12.8) |
|  | BCP | 53 (10.5) | 11 (7.1) | 35 (9.3) | 99 (9.5) |

BCP, basal core phenotype; ER, estrogen receptor; HT, hormonal therapy; MRM, modified radical mastectomy; N, number; PgR, progesterone receptor; RT, radiotherapy; TNBC, triple-negative breast cancer.

^1^p<0.001, ^2^p=0.004, ^3^p=0.020, ^4^p<0.001, ^5^p=0.002,^6^p=0.022, ^7^p<0.001, ^8^p<0.001
