# Supplementary material for: Differential Response of Immunohistochemically Defined Breast Cancer Subtypes to Anthracycline-Based Adjuvant Chemotherapy with or without Paclitaxel
Source: PLoS One. 2012 Jun 5;7(6):e37946. doi: 10.1371/journal.pone.0037946 (PMC3367950; doi:10.1371/journal.pone.0037946)
Supplement: Table S3 — Selected patient and tumor characteristics according to paclitaxel or non-paclitaxel containing regimens (for subtype description see Table 2 legend in manuscript). (DOC) [file pone.0037946.s005.doc]

|  | | **Non-paclitaxel treated** | **Paclitaxel treated** | **All patients** |
| --- | --- | --- | --- | --- |
|  | | **N=156** | **N=883** | **N=1,039** |
| Age in years | Median (range) | 51 (22-78) | 53 (22-79) | 53 (22-79) |
| N of positive nodes^1^ | Median (range) | 6 (0-35) | 4 (0-54) | 4 (0-54) |
|  |  | **N (%)** | **N (%)** | **N (%)** |
| Age | <34 | 6 (3.8) | 34 (3.9) | 40 (3.8) |
|  | 34-50 | 68 (43.6) | 332 (37.6) | 400 (38.5) |
|  | >50 | 82 (52.6) | 517 (58.6) | 599 (57.7) |
| Menopausal status | Premenopausal | 81 (51.9) | 402 (45.5) | 483 (46.5) |
|  | Postmenopausal | 75 (48.1) | 481 (54.5) | 556 (53.5) |
| Type of surgery^2^ | MRM | 119 (76.3) | 593 (67.2) | 712 (68.5) |
|  | Breast conserving | 37 (23.7) | 290 (32.8) | 327 (31.5) |
| Tumor size (cm)^3^ | <2 | 57 (36.5) | 261 (29.6) | 318 (30.6) |
|  | 2-5 | 71 (45.5) | 522 (59.1) | 593 (57.1) |
|  | >5 | 28 (17.9) | 100 (11.3) | 128 (12.3) |
| Histological type | Invasive ductal | 112 (71.8) | 692 (78.4) | 804 (77.4) |
|  | Invasive lobular | 19 (12.2) | 87 (9.9) | 106 (10.2) |
|  | Mixed | 16 (10.3) | 60 (6.8) | 76 (7.3) |
|  | Other | 9 (5.8) | 44 (5.0) | 53 (5.1) |
| N of positive nodes^4^ | 0 | 2 (1.3) | 2 (0.2) | 4 (0.4) |
|  | 1-4 | 39 (25.0) | 362 (41.0) | 401 (38.6) |
|  | ≥4 | 115 (73.7) | 519 (58.8) | 634 (61.0) |
| Adjuvant HT^5^ |  | 137 (87.8) | 668 (75.7) | 805 (77.5) |
| Adjuvant RT |  | 123 (78.8) | 662 (75.0) | 785 (75.6) |
| Histological grade^6^ | 1 | 4 (2.6) | 48 (5.4) | 52 (5.0) |
|  | 2 | 88 (56.4) | 378 (42.8) | 466 (44.9) |
|  | 3 | 64 (41.0) | 455 (51.5) | 519 (50.0) |
|  | Undifferentiated | - | 2 (0.2) | 2 (0.2) |
| Ki67^7^ | <14 | 27 (17.3) | 313 (35.4) | 340 (32.7) |
|  | ≥14 | 129 (82.7) | 563 (63.8) | 692 (66.6) |
|  | Missing data | - | 7 (0.8) | 7 (0.7) |
| ER | Negative | 42 (26.9) | 239 (27.1) | 281 (27.0) |
|  | Positive | 113 (72.4) | 641 (72.6) | 754 (72.6) |
|  | Missing data | 1 (0.6) | 3 (0.3) | 4 (0.4) |
| PgR | Negative | 54 (34.6) | 289 (32.7) | 343 (33.0) |
|  | Positive | 102 (65.4) | 593 (67.2) | 695 (66.9) |
|  | Missing data | - | 1 (0.1) | 1 (0.1) |
| HER2 status | Negative | 122 (78.2) | 665 (75.3) | 787 (75.7) |
|  | Positive | 34 (21.8) | 218 (24.7) | 252 (24.3) |
| Tumor subtype^8^ | Luminal A | 16 (10.3) | 242 (27.4) | 258 (24.8) |
|  | Luminal B | 87 (55.8) | 309 (35.0) | 396 (38.1) |
|  | Luminal-HER2 | 21 (13.5) | 121 (13.7) | 142 (13.7) |
|  | HER2-enriched | 13 (8.3) | 97 (11.0) | 110 (10.6) |
|  | TNBC | 19 (12.2) | 114 (12.9) | 133 (12.8) |
|  | BCP | 11 (7.1) | 88 (10.0) | 99 (9.5) |

BCP, basal core phenotype; ER, estrogen receptor; HT, hormonal therapy; MRM, modified radical mastectomy; N, number; PgR, progesterone receptor; RT, radiotherapy; TNBC, triple-negative breast cancer.

^1^p<0.001, ^2^p=0.025, ^3^p=0.004, ^4^p<0.001, ^5^p<0.001, ^6^p=0.012, ^7^p<0.001, ^8^p<0.001
