# Supplementary material for: Differential Response of Immunohistochemically Defined Breast Cancer Subtypes to Anthracycline-Based Adjuvant Chemotherapy with or without Paclitaxel
Source: PLoS One. 2012 Jun 5;7(6):e37946. doi: 10.1371/journal.pone.0037946 (PMC3367950; doi:10.1371/journal.pone.0037946)
Supplement: Table S4 — Selected patient and tumor characteristics according to clinical trial (for subtype description see Table 2 legend in manuscript). (DOC) [file pone.0037946.s006.doc]

|  | | **HE10/97** | **HE10/00** | **All patients** |
| --- | --- | --- | --- | --- |
|  | | **N=291** | **N=748** | **N=1,039** |
| Age in years^1^ | Median (range) | 51 (22-78) | 53 (22-79) | 53 (22-79) |
| N of positive nodes^2^ | Median (range) | 7 (0-54) | 4 (0-40) | 4 (0-54) |
|  |  | **N (%)** | **N (%)** | **N (%)** |
| Age | <34 | 13 (4.5) | 27 (3.6) | 40 (3.8) |
|  | 34-50 | 126 (43.3) | 274 (36.6) | 400 (38.5) |
|  | >50 | 152 (52.2) | 447 (59.8) | 599 (57.7) |
| Menopausal status | Premenopausal | 148 (50.9) | 335 (44.8) | 483 (46.5) |
|  | Postmenopausal | 143 (49.1) | 413 (55.2) | 556 (53.5) |
| Type of surgery^3^ | MRM | 222 (76.3) | 490 (65.5) | 712 (68.5) |
|  | Breast conserving | 69 (23.7) | 258 (34.5) | 327 (31.5) |
| Tumor size (cm)^4^ | <2 | 96 (33.0) | 222 (29.7) | 318 (30.6) |
|  | 2-5 | 144 (49.5) | 449 (60.0) | 593 (57.1) |
|  | >5 | 51 (17.5) | 77 (10.3) | 128 (12.3) |
| Histology type | Invasive ductal | 212 (72.9) | 592 (79.1) | 804 (77.4) |
|  | Invasive lobular | 35 (12.0) | 71 (9.5) | 106 (10.2) |
|  | Mixed | 29 (10.0) | 47 (6.3) | 76 (7.3) |
|  | Other | 15 (5.2) | 38 (5.1) | 53 (5.1) |
| N of positive nodes^5^ | 0 | 4 (1.4) | - | 4 (0.4) |
|  | 1-4 | 62 (21.3) | 339 (45.3) | 401 (38.6) |
|  | ≥4 | 225 (77.3) | 409 (54.7) | 634 (61.0) |
| Adjuvant HT^6^ |  | 261 (89.7) | 544 (72.7) | 805 (77.5) |
| Adjuvant RT |  | 235 (80.8) | 550 (73.5) | 785 (75.6) |
| Histological grade | 1 | 12 (4.1) | 40 (5.3) | 52 (5.0) |
|  | 2 | 135 (46.4) | 331 (44.3) | 466 (44.9) |
|  | 3 | 142 (48.8) | 377 (50.4) | 519 (50.0) |
|  | Undifferentiated | 2 (0.7) | - | 2 (0.2) |
| Ki67^7^ | <14 | 49 (16.8) | 291 (38.9) | 340 (32.7) |
|  | ≥14 | 240 (82.5) | 452 (60.4) | 692 (66.6) |
|  | Missing data | 2 (0.7) | 5 (0.7) | 7 (0.7) |
| ER | Negative | 85 (29.2) | 196 (26.2) | 281 (27.0) |
|  | Positive | 205 (70.4) | 549 (73.4) | 754 (72.6) |
|  | Missing data | 1 (0.3) | 3 (0.4) | 4 (0.4) |
| PgR | Negative | 107 (36.8) | 236 (31.6) | 343 (33.0) |
|  | Positive | 184 (63.2) | 511 (68.3) | 695 (66.9) |
|  | Missing data | - | 1 (0.1) | 1 (0.1) |
| HER2 status | Negative | 217 (74.6) | 570 (76.2) | 787 (75.7) |
|  | Positive | 74 (25.4) | 178 (23.8) | 252 (24.3) |
| Tumor subtype^8^ | Luminal A | 34 (11.7) | 224 (29.9) | 258 (24.8) |
|  | Luminal B | 144 (49.5) | 252 (33.7) | 396 (38.1) |
|  | Luminal-HER2 | 42 (14.4) | 100 (13.2) | 142 (13.7) |
|  | HER2-enriched | 32 (11.0) | 78 (10.4) | 110 (10.6) |
|  | TNBC | 39 (13.4) | 94 (12.6) | 133 (12.8) |
|  | BCP | 29 (10.0) | 70 (9.4) | 99 (9.5) |

BCP, basal core phenotype; ER, estrogen receptor; HT, hormonal therapy; MRM, modified radical mastectomy; N, number; PgR, progesterone receptor; RT, radiotherapy; TNBC, triple-negative breast cancer.

^1^p=0.031, ^2^p<0.001, ^3^p<0.001, ^4^p<0.001, ^5^p<0.001, ^6^p<0.001, ^7^p<0.001, ^8^p<0.001
