# Supplementary material for: Differential Response of Immunohistochemically Defined Breast Cancer Subtypes to Anthracycline-Based Adjuvant Chemotherapy with or without Paclitaxel
Source: PLoS One. 2012 Jun 5;7(6):e37946. doi: 10.1371/journal.pone.0037946 (PMC3367950; doi:10.1371/journal.pone.0037946)
Supplement: Table S5 — Comparison of patients with and without available blocks in the two trials. (DOC) [file pone.0037946.s007.doc]

|  |  | **HE10/97** | | | **HE10/00** | | |
| --- | --- | --- | --- | --- | --- | --- | --- |
|  |  | **Block**  **available** | **No block**  **available** | **p-value** | **Block**  **available** | **No block**  **available** | **p-value** |
|  |  |
|  |  | **N=291** | **N=304** |  | **N=748** | **N=338** |  |
| Age in years | Median (range) | 51 (22-78) | 49 (24-75) | 0.095 | 53 (22-79) | 52 (24-77) | 0.14 |
| N of positive nodes | Median (range) | 7 (0-54) | 5 (0-49) | 0.065 | 4 (0-40) | 3 (0-37) | 0.012 |
|  |  | **N (%)** | **N (%)** |  | **N (%)** | **N (%)** |  |
| Age | <34 | 13 (4.5) | 27 (8.9) | 0.092 | 27 (3.6) | 19 (5.6) | 0.095 |
|  | 34-50 | 126 (43.3) | 129 (42.4) |  | 274 (36.6) | 137 (40.5) |  |
|  | >50 | 152 (52.2) | 147 (48.4) |  | 447 (59.8) | 181 (53.6) |  |
|  | Missing data | - | 1 (0.3) |  | - | 1 (0.3) |  |
| Menopausal status | Premenopausal | 148 (50.9) | 173 (56.9) | 0.16 | 335 (44.8) | 167 (49.4) | 0.17 |
|  | Postmenopausal | 143 (49.1) | 131 (43.1) |  | 413 (55.2) | 171 (50.6) |  |
| Type of surgery | MRM | 222 (76.3) | 229 (75.3) | 0.85 | 490 (65.5) | 218 (64.5) | 0.84 |
|  | Breast conserving | 69 (23.7) | 75 (24.7) |  | 258 (34.5) | 119 (35.2) |  |
|  | Missing data | - | - |  | - | 1 (0.3) |  |
| Tumor size (cm) | <2 | 96 (33.0) | 89 (29.3) | 0.11 | 222 (29.7) | 124 (36.7) | 0.038 |
|  | 2-5 | 144 (49.5) | 175 (57.6) |  | 449 (60.0) | 175 (51.8) |  |
|  | >5 | 51 (17.5) | 40 (13.2) |  | 77 (10.3) | 36 (10.7) |  |
|  | Missing data | - | - |  | - | 3 (0.9) |  |
| Histology type | Invasive ductal | 212 (72.9) | 224 (73.7) | 0.84 | 592 (79.1) | 257 (76.0) | 0.15 |
|  | Invasive lobular | 35 (12.0) | 30 (9.9) |  | 71 (9.5) | 37 (10.9) |  |
|  | Mixed | 29 (10.0) | 26 (8.6) |  | 47 (6.3) | 17 (5.0) |  |
|  | Other | 15 (5.2) | 16 (5.3) |  | 38 (5.1) | 26 (7.7) |  |
|  | Missing data | - | 8 (2.6) |  | - | 1 (0.3) |  |
| N of positive nodes | 0 | 4 (1.4) | 7 (2.3) | 0.037 | - | - | 0.003 |
|  | 1-4 | 62 (21.3) | 90 (29.6) |  | 339 (45.3) | 187 (55.3) |  |
|  | ≥4 | 225 (77.3) | 207 (68.1) |  | 409 (54.7) | 151 (44.7) |  |
| Adjuvant HT |  | 261 (89.7) | 264 (86.8) | 0.31 | 544 (72.7) | 235 (69.5) | 0.83 |
| Adjuvant RT |  | 235 (80.8) | 226 (74.3) | 0.063 | 550 (73.5) | 226 (66.9) | 0.029 |
| Histological grade | 1 | 12 (4.1) | 14 (4.6) | 0.44 | 40 (5.3) | 24 (7.1) | 0.032 |
|  | 2 | 135 (46.4) | 129 (42.4) |  | 331 (44.3) | 168 (49.7) |  |
|  | 3 | 142 (48.8) | 159 (52.3) |  | 377 (50.4) | 143 (42.3) |  |
|  | Undifferentiated | 2 (0.7) | - |  | - | 1 (0.3) |  |
|  | Missing data | - | 2 (0.7) |  | - | 2 (0.6) |  |

HT, hormonal therapy; MRM, modified radical mastectomy; N, number; RT, radiotherapy.
