# Supplementary material for: Differential Response of Immunohistochemically Defined Breast Cancer Subtypes to Anthracycline-Based Adjuvant Chemotherapy with or without Paclitaxel
Source: PLoS One. 2012 Jun 5;7(6):e37946. doi: 10.1371/journal.pone.0037946 (PMC3367950; doi:10.1371/journal.pone.0037946)
Supplement: Table S6 — Survival status for all patients. (DOC) [file pone.0037946.s008.doc]

|  | **All patients**  **N=1,039** | **HE10/97**  **N=291** | **HE10/00**  **N=748** |
| --- | --- | --- | --- |
| **Disease-free survival** |  |  |  |
| Relapses N (%) | 311 (29.9) | 107 (36.8) | 204 (27.3) |
| Events N (%) | 377 (36.3) | 130 (44.7) | 247 (33.0) |
| 5-year rate (%) | 73.1 | 69.5 | 74.6 |
| 10-year rate (%) | 60.3 | 57.2 | 60.9 |
| Range | 4.0-160.5 | 4.0-160.5 | 4.3-118.4 |
| **Overall survival** |  |  |  |
| Deaths N (%) | 275 (26.5) | 104 (35.7) | 171 (22.9) |
| 5-year rate (%) | 86.1 | 83.7 | 87.1 |
| 10-year rate (%) | 70.6 | 67.0 | 72.1 |
| Range | 4.3-160.5 | 12.1-160.5 | 4.3-121.9 |
| **Median follow-up (range)** | 105.4 (0.1-166.7) | 141.2 (7.0-166.7) | 98.9 (0.1-132.5) |
